# Anthropometric and metabolic assessment in adults with Down syndrome: the need for novel indices and tailored criteria

**DOI:** 10.55730/1300-0144.6071

**Published:** 2025-09-01

**Authors:** Selin TEKİN, Nergis BASMACI, İncilay LAY, Alper GÜRLEK

**Affiliations:** 1 Division of Endocrinology and Metabolism, Department of Internal Medicine, Faculty of Medicine, Hacettepe University, Ankara, Turkiye; 2 Division of Endocrinology and Metabolism, Department of Internal Medicine, Faculty of Medicine, Hacettepe University, Ankara, Turkiye; 3 Department of Biochemistry, Faculty of Medicine, Hacettepe University, Ankara, Turkiye; 4 Division of Endocrinology and Metabolism, Department of Internal Medicine, Faculty of Medicine, Hacettepe University, Ankara, Turkiye

**Keywords:** Down syndrome, metabolic syndrome, inflammation, obesity

## Abstract

**Background/aim:**

This study aimed to evaluate the metabolic profile of adults with Down syndrome (DS) using novel anthropometric and metabolic indices, while also highlighting the limitations of traditional obesity assessment methods compared with the non-DS population.

**Materials and methods:**

This cross-sectional study included 22 adults with DS and 28 age- and sex-matched non-DS controls. Anthropometric measurements, including BMI, waist circumference (WC), and hip circumference (HC), were recorded alongside novel indices such as waist-to-height ratio (WHtR), body adiposity index (BAI), A body shape index (ABSI), and visceral adiposity index (VAI). Metabolic parameters (fasting plasma glucose, HbA1c, lipid profiles, HOMA-IR) and CBC-derived inflammation indices (NLR, PLR, MHR, NHR, LHR, PHR, SII, AISI, SIRI) were evaluated.

**Results:**

Despite comparable BMI, individuals with DS exhibited significantly higher WHtR and BAI values (p < 0.001), suggesting greater central adiposity. Interestingly, the prevalence of metabolic syndrome was lower in the DS group (9.1%), and diastolic blood pressure was significantly lower as well. WHtR and BAI demonstrated stronger associations with metabolic markers than BMI. Among inflammatory indices, PLR was uniquely elevated in individuals with DS (p = 0.038), while VAI showed strong correlations with both.

**Conclusion:**

Adults with DS display distinct cardiometabolic profiles that are poorly captured by traditional indices. Novel markers such as WHtR, BAI, and VAI offer enhanced insight into metabolic risk, whereas PLR emerges as a potential inflammatory biomarker. These findings underscore the need for population- and sex-specific criteria in evaluating metabolic health in DS. Tailored diagnostic models may improve risk stratification and clinical outcomes for this vulnerable group.

## 1. Introduction

Down syndrome (DS), or trisomy 21, is the most common chromosomal disorder, caused by an extra copy of chromosome 21 (HSA21) and involving dysregulation of key genes within the Down syndrome critical region (DSCR) and other loci, contributing to neurodevelopmental and metabolic abnormalities [1–7]. Advances in medical care have increased the life expectancy of individuals with DS, resulting in a growing adult population that exhibits distinct metabolic and immune dysfunctions, including oxidative stress, premature aging, obesity, and dyslipidemia [8–14]. Weight management remains a major concern, as early-life feeding difficulties and a high prevalence of obesity often persist into adulthood [15–18]. These alterations are associated with factors such as low metabolic rate, hypotonia, systemic inflammation, reduced activity levels, and telomere shortening [11, 13, 19–21].

Metabolic syndrome (MetS) is characterized by a cluster of conditions that increase the risk of atherosclerotic cardiovascular diseases, including myocardial infarction, stroke, peripheral artery disease, insulin resistance, and type 2 diabetes mellitus [22]. Abdominal obesity and insulin resistance are key drivers of MetS. In adults with DS, abdominal obesity has been associated with higher rates of weight-related disorders, dyslipidemia, and impaired glucose metabolism [23]. Interestingly, despite these risk factors, the prevalence of MetS among individuals with DS appears lower than in the general population, possibly due to lower blood pressure and more favorable lipid profiles [24, 25]. Moreover, subclinical atherosclerosis and cardiovascular events remain rare in this group [12, 26, 27]. Studies using carotid intima-media thickness and pulse wave velocity have confirmed no significant increase in subclinical vascular damage among adults with DS compared with healthy controls, despite similar cardiovascular risk profiles [12]. However, limited sample sizes and predominantly cross-sectional study designs preclude definitive conclusions.

Given these unique metabolic and cardiovascular characteristics, relying solely on conventional anthropometric indices such as body mass index (BMI) and waist circumference (WC) to assess obesity and metabolic syndrome in individuals with DS may lead to inaccurate evaluations. It is crucial to incorporate novel inflammatory and anthropometric indices tailored to the DS population to better capture their specific metabolic and inflammatory profiles.

## 2. Materials and methods

### 2.1. Study design and participants

We conducted a cross-sectional study involving 22 adults with DS and 28 non-DS controls who were admitted to the Division of Endocrinology and Metabolism, Department of Internal Medicine, Hacettepe University School of Medicine, Ankara, Türkiye for endocrinological evaluation between October 2024 and January 2025. Ethical approval for the study was obtained from the relevant ethics committee (approval number: 2024/18–18).

The inclusion criteria were being over 18 years of age and having DS documented by karyotype. Control participants were age and sex matched non-DS individuals. Control participants were excluded if they had (i) acute or chronic inflammatory disease, (ii) active malignancy, (iii) chronic renal or hepatic failure, (iv) a history of congenital heart disease with current hemodynamic compromise, or (v) were using medications with direct effects on lipid or glucose metabolism (e.g., statins, metformin, systemic corticosteroids). Women who were pregnant or lactating were also excluded.

### 2.2. Measurements

Demographic and medical information of each participant was recorded (age, sex, medical history, disease duration, and medications used). Blood pressure (mmHg), height (cm), body weight (kg), waist circumference (cm), and hip circumference (cm) of all participants were recorded. BMI was calculated using the formula weight/height^2^ and expressed in kg/m^2^. WC was measured with a tape measure at the midpoint between the lower rib and the iliac crest while participants were standing. Hip circumference (HC) was measured parallel to the ground at the level of the greater trochanter of the femur. BMI was used as the criterion for diagnosing obesity. The body composition of the participants was evaluated using a five-segmental bioelectrical impedance analysis conducted with the Tanita Body Fat Analyzer (model BC-418; Tanita Corporation, Tokyo, Japan). The recorded measurements included body fat mass, muscle mass, fat percentage, lean body mass percentage, as well as muscle mass in the upper extremities, lower extremities, and trunk.

**Metabolic syndrome definition (IDF, 2005) [28, 64] :**

Metabolic syndrome was defined according to the 2005 International Diabetes Federation (IDF) consensus. The IDF requires central obesity (defined by waist circumference using ethnicity-specific thresholds) plus any two of the following components:

1. Triglycerides ≥ 150 mg/dL (1.7 mmol/L) or specific treatment for this lipid abnormality;
2. HDL cholesterol < 40 mg/dL (1.03 mmol/L) in males or < 50 mg/dL (1.29 mmol/L) in females, or specific treatment;
3. Blood pressure ≥ 130/85 mmHg or treatment of previously diagnosed hypertension;
4. Fasting plasma glucose ≥ 100 mg/dL (5.6 mmol/L) or a previously diagnosis of type 2 diabetes.
5. WC was measured with participants standing, at the midpoint between the lowest rib and the iliac crest, after a gentle expiration. Ethnicity-specific thresholds were applied according to the IDF consensus.

#### 2.2.1. Novel anthropometric measurements and body composition

**Waist-to-height ratio (WHtR):**

The waist-to-height ratio (WHtR) was calculated by dividing WC by height, providing a measure of central adiposity relative to body size [29].

**Body adiposity index (BAI):**

The body adiposity index (BAI) was calculated using the following formula [29]:


$$\text{BAI}=\left(\frac{\text{Hip Circumference (cm)}}{{\text{Height(m)}}^{\text{1}\text{.5}}}\right)-18$$

**A body shape index (ABSI):**

A body shape index (ABSI) was defined using the following formula [30]:


$$\text{ABSI}=\frac{\text{WC}(\text{m})}{{\text{BMI}}^{2/3}\times {\text{Height}}^{1/2}}$$

**Visceral adiposity index (VAI):**

The visceral adiposity index (VAI) was defined using the following formula [31]:


$$\begin{array}{l} \text{VAI in male}=\left(\frac{\text{WC in cms}}{39.68+(1.88\times \text{BMI})}\right)\times \left(\frac{\text{TG}}{1.03}\right)\times \left(\frac{1.31}{\text{HDL}-\text{C}}\right)\\ \text{VAI in female}=\left(\frac{\text{WC in cms}}{36.58+(1.89\times \text{BMI})}\right)\times \left(\frac{\text{TG}}{0.81}\right)\times \left(\frac{1.52}{\text{HDL}-\text{C}}\right). \end{array}$$

#### 2.2.2. Laboratory measurements

Venous blood samples were collected in the morning after an overnight fast of at least 8 h. The following biochemical and hematological parameters were analyzed: fasting plasma glucose (mg/dL), fasting insulin (μIU/mL) measured using the Human Insulin ELISA kit (Bioassay Technology Laboratory, Shanghai, China), and glycated hemoglobin A1c (HbA1c, %). Insulin resistance was assessed using the homeostatic model assessment of insulin resistance (HOMA-IR), calculated with the formula: HOMA-IR = (fasting plasma glucose × fasting insulin) / 405. Additional metabolic parameters included total cholesterol (mg/dL), triglycerides (mg/dL), HDL cholesterol (mg/dL), LDL cholesterol (mg/dL), VLDL cholesterol (mg/dL), and the total cholesterol/HDL cholesterol ratio. LDL cholesterol was directly measured using a Beckman Coulter kit (Beckman Coulter Inc., Brea, CA, USA) on the AU680 autoanalyzer. Complete blood count (CBC) parameters, creatinine (mg/dL), uric acid (mg/dL), and 25-hydroxyvitamin D (ng/mL) levels were also assessed. Thyroid function tests, including thyroid-stimulating hormone (TSH, μIU/mL), free T4 (ng/dL), and free T3 (ng/dL), were also measured. MetS was defined according to the IDF 2005 criteria.

#### 2.2.3. Inflammatory and cardiovascular risk indices

The following indices were calculated to assess systemic inflammation and cardiovascular risk:

- **Neutrophil-to-lymphocyte ratio (NLR):** neutrophil count (10^9^/L) / lymphocyte count (10^9^/L).
- **Platelet-to-lymphocyte ratio (PLR):** platelet count (10^9^/L) / lymphocyte count (10^9^/L).
- **Neutrophil-to-HDL cholesterol ratio (NHR):** neutrophil count (10^9^/L) / HDL cholesterol (mg/dL).
- **Monocyte-to-HDL cholesterol ratio (MHR):** monocyte count (10^9^/L) / HDL cholesterol (mg/dL).
- **Lymphocyte-to-HDL cholesterol ratio (LHR):** lymphocyte count (10^9^/L) / HDL cholesterol (mg/dL).
- **Platelet-to-HDL cholesterol ratio (PHR):** platelet count (10^9^/L) / HDL cholesterol (mg/dL).
- **Systemic inflammatory response index (SIRI):** (monocyte count × neutrophil count) / lymphocyte count (10^9^/L).
- **Systemic immune-inflammation index (SII):** (platelet count × neutrophil count) / lymphocyte count (10^9^/L).
- **Aggregate index of systemic inflammation (AISI):** (neutrophil count × monocyte count × platelet count) / lymphocyte count (10^9^/L).
- **Homeostasis model assessment of insulin resistance (HOMA-IR):** (fasting insulin (μIU/mL) × fasting glucose (mg/dL)) / 405.
- **Triglycerides-to-HDL cholesterol ratio (TG/HDL):** triglycerides (mg/dL) / HDL cholesterol (mg/dL).
- **Non-HDL cholesterol (non-HDL-C):** total cholesterol (mg/dL) − HDL cholesterol (mg/dL).

### 2.3. Statistical analysis

All statistical analyses were performed using SPSS software version 25.0 (IBM Corp., Armonk, NY, USA). Continuous variables were tested for normality using the Shapiro–Wilk test. Variables with a normal distribution were expressed as mean ± standard deviation (SD), while nonnormally distributed variables were expressed as median (minimum–maximum). Categorical variables were presented as frequencies and percentages. The independent-samples t-test was used to compare normally distributed continuous variables between groups, and the Mann–Whitney U test was applied for nonnormally distributed variables. Categorical variables were analyzed using the chi-square test or Fisher’s exact test, as appropriate. Correlations between anthropometric indices (e.g., BMI, waist-to-height ratio) and metabolic parameters (e.g., fasting plasma glucose, HbA1c, systolic blood pressure, diastolic blood pressure, lipid profile) were evaluated using Pearson’s correlation coefficient for normally distributed variables and Spearman’s rank correlation coefficient for nonnormally distributed variables. Statistical significance was set at p < 0.05 for all analyses. All results are presented in tables, and detailed comparisons of variables and their correlations are provided in Tables 1–5.

## 3. Results

### 3.1. Study population

A total of 22 individuals with DS (12 males, 10 females) and 28 non-DS controls (13 males, 15 females) were enrolled in the study. Sex distribution did not differ significantly between groups (p = 0.569). The mean age was 28.3 ± 8.9 years in the DS group and 31.4 ± 7.2 years in the control group, with no statistically significant difference between the groups (p = 0.21). Among individuals with DS, the most common comorbidities included hypothyroidism (n = 16), congenital or acquired cardiac anomalies (n = 8), hearing impairment (n = 3), visual disturbances such as cataracts (n = 3), epilepsy (n = 3), obstructive sleep apnea syndrome (n = 3), and inflammatory bowel disease (n = 4), two of whom had a history of hemorrhoidectomy. Additionally, single cases of celiac disease and osteoporosis were recorded (Table 1). None of the participants had a diagnosis of diabetes mellitus. One individual in the DS cohort and one in the non-DS cohort met the criteria for prediabetes based on impaired fasting glucose, but no participants in either group had diabetes. Mean HbA1c levels were comparable between groups (DS: 5.26% ± 0.4% versus controls: 5.29% ± 0.3%; p = 0.71).

### 3.2. Anthropometric characteristics and obesity

The mean body weight of individuals with DS was significantly lower compared with controls (63.25 kg [56.6–83.1] versus 83.95 kg [73.5–93.1]; p = 0.003). Similarly, the mean height was markedly reduced in the DS group (148.1 ± 10.1 cm) compared with the control group (168.5 ± 10.2 cm; p < 0.001). However, no significant difference was observed in BMI between the groups (DS: 30.3 ± 8.3 kg/m^2^ versus controls: 28.3 ± 4.1 kg/m^2^; p = 0.35). Further anthropometric and clinical characteristics are detailed in Table 1. In the DS group, 27% of individuals had normal weight, 23% were overweight, and the prevalence of class I, class II, and morbid obesity was 23%, 14%, and 14%, respectively. In contrast, among controls, 18% had normal weight, 43% were overweight, 36% had class I obesity, and 4% had class II obesity. The WHtR was significantly higher in the DS group compared with controls (p < 0.001). Body fat percentage and lean body mass percentage did not differ significantly between the groups (p = 0.70) (Table 1).

### 3.3. Metabolic parameters

Diastolic blood pressure was significantly lower in individuals with DS compared with controls (p = 0.04), while systolic blood pressure showed no significant difference (Table 2). Fasting glucose, insulin, HbA1c, HOMA-IR, lipid profile, and transaminase levels were comparable between groups (Table 2). The prevalence of metabolic syndrome (MetS) was significantly lower in the DS group (9.1%) compared with controls (21.4%) (p = 0.001) (Table 1). Among the DS cohort, only two patients met the diagnostic criteria for metabolic syndrome. Due to the very small sample size, formal statistical comparisons with controls were not performed, as such analyses could be misleading. Instead, the clinical and biochemical characteristics of these two patients are presented individually in Table 3 (labeled as Patient 1 and Patient 2) to provide a descriptive overview of their profiles.

### 3.4. Abdominal obesity and associated findings

Adults with DS and non-DS controls had comparable BMI, yet individuals with DS exhibited significantly greater central adiposity, as indicated by higher WHtR (0.64 ± 0.12 versus 0.52 ± 0.06; p < 0.001) and body adiposity index (42.3 ± 10.4 versus 30.5 ± 4.7; p < 0.001). Based on WHtR, the prevalence of abdominal obesity was 86.3% in the DS group and 78.5% in the control group. Among those with abdominal obesity, individuals with DS were younger than controls (p = 0.03). Additionally, the weight (66.3 [44–98] kg) and height (147 ± 10 cm) of individuals with DS and abdominal obesity were significantly lower than those of the control group (85.5 [73–97] kg, 167.9 ± 3.2 cm), with p = 0.002 and p < 0.001, respectively. The WHtR of individuals with DS with abdominal obesity was also higher than that of the control group (p < 0.001). Although the glucose, lipid, and blood pressure profiles of individuals with DS with abdominal obesity were better than those of the control group, the differences were not statistically significant (Table 4).

### 3.5. Novel anthropometric indices

The VAI values were similar between the DS and control groups (p = 0.96). In contrast, the BAI was significantly higher in the DS group compared with the control group (p < 0.001), and the ABSI was also elevated in the DS group (p = 0.001) (Table 6). Within the DS group, the BAI was significantly higher in women than in men (p = 0.001), while the VAI and ABSI showed no significant sex differences (Table 5).

### 3.6. Novel CBC-Derived inflammatory markers

Among inflammatory markers, PLR was elevated in DS (132.7 [107–144] versus 106.2 [83–124]; p = 0.038). The other indices, including NLR, PHR, MHR, NHR, SII, SIRI, and AISI, did not show significant differences between the two groups (Table 6).

### 3.7. Correlation analysis

Correlation analyses in the DS group demonstrated that WHtR correlated positively with HbA1c (r = 0.55, p = 0.008), systolic blood pressure (r = 0.80, p < 0.001), diastolic blood pressure (r = 0.75, p < 0.001), waist circumference (r = 0.94, p < 0.001), and triglycerides (r = 0.37, p = 0.087). Similarly, BAI correlated with HbA1c (r = 0.48, p = 0.025), systolic blood pressure (r = 0.70, p < 0.001), diastolic blood pressure (r = 0.68, p = 0.001), and triglycerides (r = 0.67, p = 0.001). In contrast, BMI showed weaker associations (HbA1c: r = 0.47, p = 0.029; SBP: r = 0.85, p < 0.001; DBP: r = 0.76, p < 0.001; TG: r = 0.88, p < 0.001), while ABSI exhibited minimal or inconsistent correlations (all p > 0.05 except DBP: r = 0.43, p = 0.045). VAI correlated with diastolic blood pressure (r = 0.43, p = 0.046), triglycerides (r = 0.66, p = 0.001), HDL cholesterol (r = 0.51, p = 0.015), and PLR (r = 0.43, p = 0.041), highlighting its relevance as a combined metabolic and inflammatory marker.

Among inflammatory indices, BAI correlated positively with PLR (r = 0.49, p = 0.019), and VAI correlated with PLR (r = 0.43, p = 0.041) and HDL-related ratios (Table 7). In the DS group, BMI and WHtR showed no significant correlations with any of the inflammation indices (all p > 0.05). The body adiposity index was positively correlated with PLR (r = 0.49, p = 0.019), while ABSI was correlated with PHR (r = 0.45, p = 0.041). Additionally, the VAI was associated with multiple inflammation indices, including PLR (r = 0.43, p = 0.046), PHR (r = 0.44, p = 0.041), MHR (r = 0.51, p = 0.015), and NHR (r = 0.66, p = 0.001). The correlation patterns between anthropometric indices and inflammation indices also varied by sex, as demonstrated in Table 8.

## 4. Discussion

This study provides novel insights into the distinct metabolic, anthropometric, and inflammatory profiles of adults with DS, while also highlighting the limitations of conventional indices and the potential utility of novel parameters.

Although BMI remains the most widely used index for obesity assessment, it is neither accurate nor reliable as an indicator of adipose tissue mass at the individual level [32–36]. Moreover, its predictive value for metabolic dysfunction varies across populations with diverse ethnic backgrounds and body proportions [41,42]. These limitations are particularly evident in DS, where short stature and altered body composition compromise the validity of BMI [37–40]. Indeed, despite comparable BMI values between DS and controls in our cohort, DS participants demonstrated significantly greater central adiposity, reflected by elevated WHtR.

Paradoxically, despite increased abdominal adiposity, as shown by elevated WHtR and BAI, individuals with DS exhibited a lower prevalence of MetS when the IDF criteria were applied [28]. These findings support growing evidence that DS is characterized by a unique cardiometabolic risk pattern [12, 14, 24–27, 43]. Importantly, while metabolic parameters were largely comparable between groups, individuals with DS maintained lower diastolic blood pressure and a lower MetS prevalence despite increased abdominal obesity, suggesting a decoupling of obesity from metabolic dysfunction, as previously reported [14, 24, 25]. The IDF definition mandates central obesity [28]; however, this requirement may be problematic in DS due to shorter stature and atypical fat distribution patterns, which challenge the validity of absolute waist circumference thresholds. In our cohort, only two participants with DS fulfilled the IDF definition, precluding sensitivity analyses with alternative frameworks. Conceptually, however, definitions not mandating waist circumference (e.g., ATP III) or incorporating height-normalized measures might classify a greater proportion of individuals with DS as having MetS, leading to higher prevalence estimates and potentially more accurate risk identification.

These findings, coupled with the stronger associations observed between WHtR and BAI and metabolic markers, emphasize the need for DS-specific or adapted diagnostic criteria. Although ABSI was elevated in DS compared with controls, its predictive utility for abdominal obesity and metabolic risk appeared limited, consistent with prior reports suggesting weaker applicability than WHtR or BAI [30, 64].

Beyond anthropometric indices, this study provides the first evidence on the performance of CBC-derived markers (e.g., SIRI, AISI, and PHR) in DS. These indices were selected because prior studies have demonstrated their utility in reflecting systemic inflammation and cardiometabolic risk in obese and metabolic syndrome populations [44–46,55–57]. In particular, SII, SIRI, and AISI have been associated with insulin resistance, cardiovascular risk, and the severity of metabolic syndrome in non-DS cohorts [46, 55–57]. Platelet-based ratios (e.g., PHR, PLR) were also included as emerging markers that may capture platelet-driven immune dysregulation, which could be particularly relevant in DS given the known hematological alterations in this population. Moreover, because traditional anthropometric and metabolic indices may insufficiently capture the distinct pathophysiological features of DS, the inclusion of these novel CBC-derived inflammation indices aimed to provide a broader and more sensitive assessment of systemic inflammation in this unique population. This approach is particularly justified, as individuals with DS exhibit chronic low-grade inflammation, altered immune cell distribution, and hematological peculiarities, which may not be adequately reflected by conventional indices [46].

Our results suggest a distinct pattern in DS, in which PLR was elevated, but other indices (NLR, MLR, and SII) did not differ significantly from controls. In our analyses, VAI was associated with the greatest number of CBC-derived inflammatory indices, suggesting that visceral adiposity interacts broadly with systemic inflammatory pathways in DS. This supports the potential role of VAI as a more integrative marker of cardiometabolic risk than conventional anthropometric measures.

Furthermore, exploratory within-group analyses revealed sex-specific correlations between inflammatory indices and metabolic markers, reinforcing the importance of sex-tailored assessments [46–50]. This contrasts with non-DS populations, where inflammatory indices more consistently associate with insulin resistance and broader metabolic dysfunctions [51–57]. Nevertheless, the limited sample size, especially in sex-stratified analyses, restricts generalizability and warrants replication in larger multicenter cohorts.

Additional factors must be considered when interpreting these metabolic and inflammatory differences. Hypothyroidism, present in 72.7% of our DS cohort, is known to influence lipid profiles, body composition, insulin sensitivity, and inflammatory markers [58, 59]. Its high prevalence may have contributed to the observed patterns; however, the small sample size precluded robust stratified analyses. Likewise, elevated alkaline phosphatase (ALP) and gamma-glutamyl transferase (GGT) levels in participants with DS raise the possibility of subclinical liver involvement, given the increased risk of metabolic associated fatty liver disease (MAFLD), autoimmune hepatobiliary disorders, and drug-related hepatotoxicity in this population [60]. Elevated ALP levels may also reflect altered bone metabolism, which is common in DS [61]. Without hepatic imaging or ALP isoenzyme analysis, the relative contribution of liver versus bone sources remains uncertain. Finally, chronic use of antiepileptic and psychotropic medications, both known to adversely affect weight, insulin sensitivity, and lipid metabolism [62, 63], may also have shaped the observed metabolic phenotype.

Taken together, our findings suggest that the interplay between adiposity, inflammation, and metabolism in DS diverges fundamentally from that in the general population. These insights underscore the inadequacy of conventional anthropometric and inflammatory markers and highlight the need for DS-specific diagnostic frameworks that integrate anthropometric, biochemical, and hematological markers to improve risk stratification and guide clinical management.

## 5. Limitations and future directions

The small sample size, single-center design, reliance on TANITA for body composition analysis, and underpowered sex-stratified subgroups limit the generalizability of our findings. The low number of outcome events further restricted robust multivariable adjustments and interaction testing. Accordingly, sex-specific associations should be considered preliminary and require confirmation in larger studies. Metabolic syndrome thresholds, including the IDF waist-circumference cut-offs, have not been validated in DS, which may have led to underestimation of prevalence and should prompt cautious interpretation of between-group comparisons. Further large-scale, multicenter studies are required to validate these results and explore the underlying mechanisms driving the distinct cardiometabolic and inflammatory profiles observed in DS. Such investigations may help refine diagnostic strategies and improve risk stratification in this population.

## 6. Conclusions

This study demonstrates that adults with DS exhibit a distinct cardiometabolic profile, characterized by central adiposity, altered inflammatory patterns, and frequent endocrine and hepatic comorbidities. Conventional indices such as BMI and standard metabolic syndrome definitions are insufficient to capture this risk, whereas novel markers, including WHtR, BAI, and VAI, showed stronger associations with metabolic alterations. Additionally, this is the first study to evaluate CBC-derived indices (SIRI, AISI, PLR, etc.) in conjunction with novel anthropometric markers (WHtR, BAI, VAI, etc.) in adults with DS, revealing unique inflammatory and metabolic signatures. Among these, platelet-based indices emerged as the most prominent, suggesting a key role of platelet dynamics in the interaction between metabolism and inflammation. Collectively, these findings emphasize the need for DS-specific assessment frameworks that integrate anthropometric, biochemical, and hematological parameters to improve early detection and enhance risk stratification. Larger studies are warranted to validate these results and clarify the mechanisms underlying the distinctive metabolic patterns observed in DS.

## Figures and Tables

**Table 1:** Demographic features of the participants

	DS (n=22)	Non-DS controls (n=28)	P

**Age (years)**	28.3± 8.9	31.4±7.2	0.21

**Gender (F/M)**	10/12	15/13	0.56

**Average weight (kg)**	63.25 (56.6–83.1)	83.95 (73.5–93.1)	**0.003**

**Average height (cm)**	148.1±10.1	168.5±10.2	**< 0.001**

**BMI (kg/m** **^2^** **)**	30.3±8.3	28.3±4.1	0.35

**WC (cm)**	98 (82.7–110)	88 (84–97)	0.16

**HC (cm)**	108 (101–117)	108 (100.7–110)	0.73

**WHtR**	0,64±0,12	0,52±0,06	**<0.001**

**Body fat percentage (%)**	21.7±11.1	28.1±8.05	0.68

**Lean body percentage (%)**	71.1±12.4	71.8±8	0.81

**Classifications according to the BMI n(%)**			
*Normal weight*	6 (27)	5 (18)	
*Overweight*	5 (23)	12 (43)	
*Class-1 obesity*	5 (23)	10 (36)	
*Class-2 obesity*	3 (14)	1 (4)	
*Morbid obesity*	3 (14)	0 (0)	

**Comorbidities**	*Hypothyroidism (n=16)*	na	
	*Cardiac pathologies (n=8)*		
	*Hearing loss (n=3)*		
	*Visual problems (n=3)*		
	*Epilepsy (n=3)*		
	*OSAS (n=3)*		
	*Inflamatory bowel disease (n=4)*		
	*Celiac disease (n=1)*		
	*Osteoporosis (n=1)*		

**Medicines**	*Levothyroxine (n=12)*	na	
	*Anti-psychotic drugs (n=3)*		
	*Anti-epileptic drugs (n=2)*		

**DS:** Down Syndrome, **BMI:** Body mass index, **WC:** Waist circumference, **HC:** Hip circumference, **WHtR:** Waist-to-height ratio.

**Na**: non-available **Normal weight:** BMI: 18.5–24.9 kg/m^2^, **Overweight:** BMI: 25.0–29.9 kg/m^2^,**Class-1 obesity:** BMI: 30–34.9 kg/m

**Class-2 obesity:** BMI: 35.0–39.9 kg/m^2^, **Morbid obesity:** BMI: >40 kg/m^2^

Data are presented as mean ± standard deviation (SD), median (min-max), or number (percentage, %), as appropriate.

**Table 2:** Laboratory measurements and vital signs of participants

	DS (n=22)	Non-DS controls (n=28)	P
**Systolic blood pressure (mmHg)**	117±6.2	119±6.2	0.25
**Diastolic blood pressure (mmHg)**	73±4.4	76±5.2	**0.04**
**Fasting blood glucose (mg/dL)**	85.5±7.5	85.6±9.4	0.83
**Fasting insülin (mU/mL)**	11.5 (8–15)	17.02 (11–44)	0.06
**Hba1c (%)**	5.26±0.4	5.29±0.3	0.71
**HOMA-IR**	2.53 (1.8–3.2)	3.13 (2.7–9.4)	0.08
**Total cholesterol (mg/dL)**	177±4.32	189±38.3	0.22
**Triglyceride (mg/dL)**	100.1±32.3	106.6±45.8	0.71
**HDL (mg/dL)**	51.6±17.2	51.6±8.9	0.56
**LDL (mg/dL)**	115.5±25.5	119.4±28.2	0.75
**Non-HDL (mg/dL)**	125±31	136.7±37.1	0.32
**ALT (U/L)**	21.3±5.48	26.6±22.5	0.62
**AST (U/L)**	21.95±3.9	22.5±9.36	0.53
**ALP (U/L)**	84.9±25.9	64.5±20.3	**0.008**
**GGT (U/L)**	32.31±31	24.1±20.63	**0.021**
**Calcium (mg/dL)**	9.38±0.35	9.55±1.24	0.2
**Phosphorus (mg/dL)**	3.71±0.46	3.56±0.50	0.81
**Uric acide (mg/dL)**	6.3±1.1	5.6±1.6	**0.07**
**PTH (pg/mL)**	44.1±17.4	42.6±20.07	0.51
**TSH (mIU/L)**	3.98±2.7	1.89±0.8	**0.003**
**Hemoglobin (gr/dL)**	15.1±1.2	14.05±1.4	**0.008**
**WBC (x10** **^9^** **/L)**	7.03	6.38	0.403
**Lymphocyte (x10** **^9^** **/L)**	2.28±0.68	2.55±0.71	0.18
**Neutrophile (x10** **^9^** **/L)**	4.10±3.71	3.57±0.74	0.46
**Monocyte (x10** **^9^** **/L)**	0.48±0.16	0.44±0.1	0.43
**Platelet (x10** **^9^** **/L)**	279.5±82	262.2±42	0.35

**DS:** Down Syndrome, **HOMA-IR:** Homeostasis model assessment for insulin resistance, **HDL:** High density lipoprotein

**LDL:** Low density lipoprotein, **PTH:** Parathormon, **TSH:** Thyroid stimulating hormone, **WBC:** White blood cell

Data are presented as mean ± standard deviation (SD), median (min-max), or number (percentage, %), as appropriate.

**Table 3:** Clinical and biochemical features of DS patients with MetS

	Patient 1	Patient 2
**Age (yrs)**	30	31
**Weight (kg)**	78	79
**Height (cm)**	143	149
**BMI (kg/m** **^2^** **)**	35.5	35.5
**WC (cm)**	115	115
**HC (cm)**	127.5	127
**WHtR**	0.77	0.77
**Fasting plasma glucose (mg/dL)**	95	95
**Fasting insülin (mU/mL)**	12.9	13
**Hba1c (%)**	5.9	5.9
**Total cholesterol (mg/dL)**	175	176
**Triglyceride (mg/dL)**	130	128
**HDL (mg/dL)**	41.5	42
**LDL (mg/dL)**	126	125
**Non-HDL (mg/dL)**	133.5	134
**Systolic blood pressure (mmHg)**	120	120
**Diastolic blood pressure (mmHg)**	75	76

**WHtR:** Waist-to-height ratio, **WC:** Waist circumference, **HC:** Hip circumference

**HDL:** High density lipoprotein, **LDL:** Low density lipoprotein

**Table 4:** Comparison of subjects with abdominal obesity

	DS (n=22)		P	Non-DS controls (n=28)		P*
	Obese	Non-obese		Obese	Non-obese	
**Age (yrs.)**	27.8±8.6	36.3±10.1	0.08	32.2±8	27.6±4.5	**0.03**
**Weight (kg)**	66.3 (44–98)	43.5 (35.6–53.6)	**0.005**	85.5 (73–97)	69.9 (64–71)	**0.002**
**Height (cm)**	147±10	149.6±11.8	0.65	167.9±3.2	171.8±13.4	**<0.001**
**WHtR**	0.67±0.11	0.45±0.04	**0.001**	0.55±0.04	0.44±0.03	**<0.001**
**FBG (mg/dL)**	85.6±1.8	85.3±0.8	0.71	87.4±1.8	77±3.9	0.28
**Fasting insülin (mU/mL)**	18.2±3.9	7.7±4	0.22	30.2±5.2	14.9±1.6	0.17
**Hba1c (%)**	5.3±0.08	5±0.35	0.58	5.2±0.07	5.2±0.1	0.7
**HOMA-IR**	3.8±0.8	1.6±0.8	0.22	6.5±1.1	2.7±0.1	0.14
**TC (mg/dL)**	178±10.5	170.3±15	1	185.6±8.9	199±14.9	0.46
**Triglyceride (mg/dL)**	105.3±7.1	67.3±8.9	**0.05**	110.2±10.7	100,1±18.7	0.97
**HDL (mg/dL)**	50.2±4.1	60.6±5.2	0.1	49.2±2.06	56.8±2.8	0.47
**LDL (mg/dL)**	117.5±6.09	103±8.2	0.46	116.9±6.6	123.3±11.3	0.92
**Non-HDL (mg/dL)**	127.4±7.4	109.6±10.5	0.46	135.8±8.8	142.3±15.6	0.56

**DS:** Down Syndrome, **WHtR:** Waist-to-height ratio, **FBG:** Fasting blood glucose, **HOMA-IR:** Homeostasis model assessment for insulin resistance

**TC:** Total cholesterol, **HDL:** High density lipoprotein, **LDL:** Low density lipoproteinP*: Comparison of DS and non-DS patients with abdominal obesity

Data are presented as mean ± standard deviation (SD), median (min-max), or number (percentage, %), as appropriate.

**Table 5:** Anthropometric indices of participants

	DS (n=22)				Non-DS controls (n=28)				P**
	F (n=10)	M (n=12)	P*	Total	F (n=15)	M (n=13)	P*	Total	
**BMI**	33.25±8.3	27.5±7.3	0.09	30.2±8.1	28.6±4.3	27.6±3.9	0.27	28.1±4.1	0.41
**WHtR**	0.69±0.1	059±0.1	0.12	0.64±0.1	0.52±0.06	0.52±0.06	0.96	0.52±0.06	**0.001**
**ABSI**	0.79±0.06	0,82±0,06	0.34	0,81±0,06	0.72±0.04	0.76±0.06	**0.046**	0,74±0,05	**0.001**
**BAI**	49.3±8.4	35.9±7.6	**0.001**	42.3±10.4	32.7±3.8	28±4.8	**0.006**	30.5±4.7	**<0.001**
**VAI**	2.63 (2.2–4.2)	3.25(2.2–4.2)	0.67	2.7(1.8–4.6)	3.06(1.8–4.6)	2.56(1.8–4.0)	0.68	2.98(2.0–4.8)	0.96

**DS:** Down Syndrome, **F:** Female, **M:** Male, **BMI:** Body mass index, **WHtR:** Waist-to-height ratio **ABSI:** A body shape index, **BAI:** Body adiposity index, **VAI:** Visceral adiposity index

P*: Comparison between female and male patients

P**: Comparison between DS and non-DS subjects

Data are presented as mean ± standard deviation (SD), median (min-max), or number (percentage, %), as appropriate.

**Table 6:** Inflammation indices of participants

	DS (n=22)	Non-DS controls (n=28)	P
**NLR**	1.37 (1.1–2.1)	1.53 (1.1–2.0)	0.43
**PLR**	132.7 (107–144)	106.2 (83–124)	**0.038**
**NHR**	90.6±19	72.6±3.9	0.67
**LHR**	47.5±17	52.3±20	0.39
**PHR**	5956±2716	5323±1653	0.50
**MHR**	10.5±5.2	8.9±3.1	0.35
**SIRI**	659 (354–1311)	566 (462–847)	0.80
**SII**	407.7 (273–596)	338.5 (287–487)	0.42
**AISI**	218.1(86–390)	152.3 (115–209)	0.44

**DS:** Down Syndrome, **NLR:** Neutrophil-to-Lymphocyte Ratio **PLR:** Platelet-to-Lymphocyte Ratio

**NHR:** Neutrophil-to- Hemoglobin Ratio, **LHR:** Lymphocyte-to-HDL Ratio

**PHR:** Platelet -to-Hemoglobin Ratio, **MHR:** Monocyte-to-Hemoglobin Ratio

**SIRI:** Systemic Inflammatory Response Index **SII:** Systemic Immune-Inflammation Index

**AISI:** Aggregate Index of Systemic Inflammation

Data are presented as mean ± standard deviation (SD), median (min-max), or number (percentage, %), as appropriate.

**Table 7:** Spearman’s rank correlations between anthropometric indices and metabolic parameters in DS

	FBG		Hba1c		HOMA		SBP		DBP		WC		TG		HDL		TG/HDL	
	r	p	r	p	r	p	r	p	r	p	r	p	r	p	r	p	r	p
**BMI**	0.382	**0.080**	0.465	**0.029**	0.073	0.746	0.850	**<0.001**	0.761	**<0.001**	0.876	**<0.001**	0.342	0.119	−0.052	0.819	0.152	0.500
**WHtR**	0.473	0.026	0.550	**0.008**	−0.02	0.917	0.799	**<0.001**	0.746	**<0.001**	0.939	**<0.001**	0.373	0.087	−0,123	0.586	0.186	0.407
**BAI**	0.316	0.152	0.476	**0.025**	−0.228	0.308	0.695	**<0.001**	0.677	**0.001**	0.669	**0.001**	0.278	0.210	0.100	0.652	0.068	0.764
**ABSI**	0.340	0.132	0.287	0.208	−0.027	0.908	0.069	0.765	0.121	0.601	0.430	0.052	0.212	0.356	−0.405	0.068	0.271	0.235
**VAI**	0.011	0.962	0.120	0.594	0.009	0.969	0.416	0.054	−0.028	0.903	0.432	**0.045**	0.814	**<0.001**	−0.563	**0.006**	0.955	**<0.001**

**FBG:** Fasting blood glucose, **SBP:** Systolic blood pressure, **DBP:** Diastolic blood pressure, **WC:** Waist circumference, **TG:** Triglyceride, **HDL:** High density lipoprotein, **LDL:** Low density lipoprotein

**BMI:** Body mass index, **WHtR:** Waist-to-height ratio, **BAI:** Body adiposity index, **ABSI:** A body shape index, **VAI:** Visceral adiposity index

**Table 8:** Correlations between anthropometric and inflammation of indices in DS

	NLR		PLR		NHR		LHR		PHR		MHR		SII		SIRI		AISI	
	r	p	r	p	r	p	r	p	r	p	r	p	r	p	r	p	r	p
**BMI**	0.169	0.452	0.181	0.420	0.253	0.257	0.090	0.691	0.190	0.396	0.049	0.828	0.224	0.310	0.146	0.517	0.185	0.409
**WHtR**	0.191	0.395	0.280	0.207	0.315	0.153	0.140	0.536	0.341	0.120	0.099	0.661	0.277	0.212	0.168	0.456	0.231	0.302
**BAI**	0.335	0.127	0.494	**0.019**	0.366	0.094	−0.142	0.527	0.292	0.187	0.038	0.868	0.415	0.055	0.309	0.161	0.366	0.094
**ABSI**	−0.108	0.642	0.105	0.650	0.048	0.836	0.345	0.125	0.449	**0.041**	0.251	0.273	−0.042	0.855	−0.107	0.645	−0.057	0.806
**VAI**	0.280	0.207	0.430	**0.046**	0.439	**0.041**	0.296	0.182	0.656	**0.001**	0.512	**0.015**	0.301	0.173	0.252	0.258	0.262	0.239

**NLR:** Neutrophil-to-Lymphocyte Ratio **PLR:** Platelet-to-Lymphocyte Ratio, **NHR:** Neutrophil-to- Hemoglobin Ratio, **LHR:** Lymphocyte-to-HDL Ratio **PHR:** Platelet -to-Hemoglobin Ratio, **MHR:** Monocyte-to-Hemoglobin Ratio, **SII:** Systemic Immune-Inflammation Index, **SIRI:** Systemic Inflammatory Response Index, **AISI:** Aggregate Index of Systemic Inflammation, **BMI:** Body mass index, **WHtR:** Waist-to-height Ratio, **BAI:** Body adiposity index, **ABSI:** A body shape index, **VAI:** Visceral adiposity index

## Data Availability

The original data generated and analyzed during this study are included in this article or in the data repositories listed in the References and may be obtained from the corresponding author upon reasonable request.
